# Promoting active participation in robot-aided rehabilitation via machine learning and impedance control

**DOI:** 10.3389/fdgth.2025.1559796

**Published:** 2025-02-21

**Authors:** Christian Tamantini, Kevin Patrice Langlois, Joris de Winter, Parham Haji Ali Mohamadi, David Beckwée, Eva Swinnen, Tom Verstraten, Bram Vanderborght, Loredana Zollo

**Affiliations:** ^1^ Research Unit of Advanced Robotics and Human-Centred Technologies, Università Campus Bio-Medico di Roma, Rome, Italy; ^2^ Institute of Cognitive Sciences and Technologies, National Research Council of Italy, Rome, Italy; ^3^ Robotics and MultiBody Mechanics Research Group, Vrije Universiteit Brussel, Brussels, Belgium; ^4^ IMEC, Leuven, Belgium; ^5^ Flanders Make, Leuven, Belgium; ^6^ Rehabilitation Research Group, Department of Physiotherapy, Human Physiology and Anatomy, Faculty of Physical Education and Physiotherapy, Vrije Universiteit Brussel, Brussels, Belgium; ^7^ Brussels Human Robotic Research Center (BruBotics), Vrije Universiteit Brussel, Brussels, Belgium; ^8^ Center for Neurosciences (C4N), Vrije Universiteit Brussel, Brussels, Belgium

**Keywords:** medical robots and systems, rehabilitation robotics, human-centered robotics, multimodal monitoring, artificial intelligence

## Abstract

**Introduction:**

Active patient participation is crucial for effective robot-assisted rehabilitation. Quantifying the user's Active Level of Participation (ALP) during therapy and developing human-robot interaction strategies that promote engagement can improve rehabilitation outcomes. However, existing methods for estimating participation are often unimodal and do not provide continuous participation assessment.

**Methods:**

This study proposes a novel approach for estimating ALP during upper-limb robot-aided rehabilitation by leveraging machine learning within a multimodal framework. The system integrates pressure sensing at the human-robot interface and muscle activity monitoring to provide a more comprehensive assessment of user participation. The estimated ALP is used to dynamically adapt task execution time, enabling an adaptive ALP-driven impedance control strategy. The proposed approach was tested in a laboratory setting using a collaborative robot equipped with the sensorized interface. A comparative analysis was conducted against a conventional impedance controller, commonly used in robot-aided rehabilitation scenarios.

**Results:**

The results demonstrated that participants using the ALP-driven impedance control exhibited significantly higher positive mechanical work and greater muscle activation compared to the control group. Additionally, subjective feedback indicated increased engagement and confidence when interacting with the adaptive system.

**Discussion:**

Closing the robot's control loop by adapting to ALP effectively enhanced human-robot interaction and motivated participants to engage more actively in their therapy. These findings suggest that ALP-driven control strategies may improve user involvement in robot-assisted rehabilitation, warranting further investigation in clinically relevant settings.

## 1 Introduction

Robotic rehabilitation platforms may play a paramount role in increasing patient active participation during therapy since they can speed up motor recovery (1). Indeed, a robotic system can include different features that aim at engaging the patient in interacting with the machine (2).

Assist-as-needed controllers are designed to provide minimal assistive forces to the patient in such a way that the robot should intervene only if the patient is not capable of performing the task autonomously (3). Furthermore, the inclusion of the patient’s intention in the control loop, i.e. to trigger the initiation of movement, results in successful clinical outcomes (4).

Although all these works stress the importance of involving and engaging patients undergoing robot-aided treatments, a methodology to continually estimate to what extent the patient is actively participating in performing the motor exercise and leveraging such metrics inside the robot control loop has not been addressed in the scientific literature. Active participation not only fosters patient engagement but also plays a pivotal role in promoting motor recovery. By actively engaging in motor exercise, patients can enhance their motor skills and improve their overall recovery outcomes (5, 6).

The Active Level of Participation (ALP) encompasses both physical and cognitive aspects (7). It includes physical workload, intention, performance, and engagement. Physical workload measures the effort exerted during rehabilitation tasks, while intention assesses the patient’s willingness to perform tasks independently, often monitored through electromyography (EMG) signals. Performance is linked to successful task completion, often gauged by tracking accuracy. Engagement and how participants perceive the interaction also influence ALP. While modeling ALP is challenging due to its complexity, these metrics show strong correlations with individuals’ ALP.

In the literature, few studies were conducted to assess the changes in the physiological response of the patient depending on his/her level of active participation. The works in (8, 9) evidenced that some distinctive features of the electroencephalogram (EEG) significantly change between active and passive walking with a lower-limb exoskeleton and in performing upper limb repetitive motions. The experiment carried out in (10) found that EEG signals can be used to extract the intention level of the subjects in response to task difficulty. Moreover, the authors evidenced that some correlations between cortical and muscle activity exist when the participants exert different levels of participation. The instrumentation used in these works is wearable, but it requires extensive calibration procedures and can be considered obtrusive, and wearing an EEG helmet is not feasible for daily rehabilitation therapy (11).

Unobtrusive wearable sensors, particularly surface EMG, have shown promise in monitoring user physiological signals. EMG sensors were used to develop a binary classifier for distinguishing between active and passive movement during a haptic device task (12). While EMG proved to be a reliable estimator of patient participation, the model was limited to predicting discrete classes and only considered EMG signals. Moreover, the literature explores various methods to estimate patients’ ALP during robot-aided rehabilitation. One approach involves analyzing the forces exchanged between humans and robots as an indicator of participation. Machine learning algorithms are utilized to deduce the perceived difficulty level, reflecting the required physical engagement (13). In (14), robot-assisted rehabilitation is conceptualized as a cooperative game, gradually reducing robot intervention as the patient’s exerted forces increase. However, relying solely on the exchanged forces may not provide an optimal estimation of participation. The analysis of interaction dynamics introduces the concept of modeling participation based on the stiffness of the targeted body area (15).

Some efforts have been made on robot control to close the loop on the patients in the so-called biocooperative systems (16). Metrics can be computed during the rehabilitation session to tune the control gains in real-time. For instance, kinematics performance as well as patient physiological parameters can be used in gait (17) and in upper limb robot-aided rehabilitation (18, 19). Recent approaches, such as the Voluntary Assist-As-Needed controller (20), adapt the assistance level provided to the user based on an EMG-driven musculoskeletal model to promote active participation. However, all these controllers aim at increasing the active involvement of the patient without explicitly quantifying the patient’s level of participation.

Therefore, current methodologies presented to estimate ALP exhibit several limitations. A significant drawback is the unimodal nature of estimation methods, where either surface EMG or interaction forces are used independently to monitor whether the subject is actively participating. While methodologies range from mathematical model-based approaches to supervised machine learning techniques aimed at binary classification of participation, these approaches fail to provide a continuous estimation of participation level. This limitation restricts the ability to capture the variability in patient ALP during therapy. However, machine learning appears to be a promising solution to conduct multimodal data integration to map user actions in discrete states. Through calibration steps, machine learning can provide a continuous estimation of ALP, enabling continuous estimation of patient involvement and facilitating the development of adaptive robotic strategies for personalized rehabilitation. Moreover, existing studies rarely propose adaptive robotic strategies that respond dynamically to the estimated state of participation. In cases where binary classification is used, the robot’s possible actions are inherently limited, reducing the scope for personalization and adaptability.

To address the limitations identified in the literature, this paper proposes a novel approach for estimating a patient’s ALP during upper-limb robot-aided rehabilitation, leveraging machine learning methodologies within a multimodal framework. Unlike previous studies that rely on unimodal signals or discrete classification, our method enables a continuous estimation of ALP, providing a more comprehensive assessment of patient engagement. To achieve this, we integrate an unobtrusive multimodal monitoring interface integrated into an end-effector rehabilitation cobot, capable of simultaneously capturing surface EMG and pressure data exchanged between the user and the robot. This approach ensures that ALP estimation does not require additional wearable sensors on the patient. Moreover, the computed ALP is used in a closed-loop adaptive impedance control strategy, namely the ALP-driven impedance control, dynamically adjusting the robot task execution time based on user engagement. As the patient’s ALP increases, the control system reduces the execution time of the task, encouraging active participation. To assess the effectiveness of the proposed approach, we conduct a comparative analysis with a conventional impedance controller, demonstrating its impact on the enrolled volunteers.

The paper is structured as follows: Section 2 provides the details of the proposed method and describes the experimental validation carried out with an end-effector robot. Section 3 presents the results obtained during the designed experiment. Lastly, Section 4 deals with the main conclusions of this work and proposes future developments.

## 2 Materials and methods

### 2.1 The proposed approach: ALP-based interaction control

**Figure 1:**
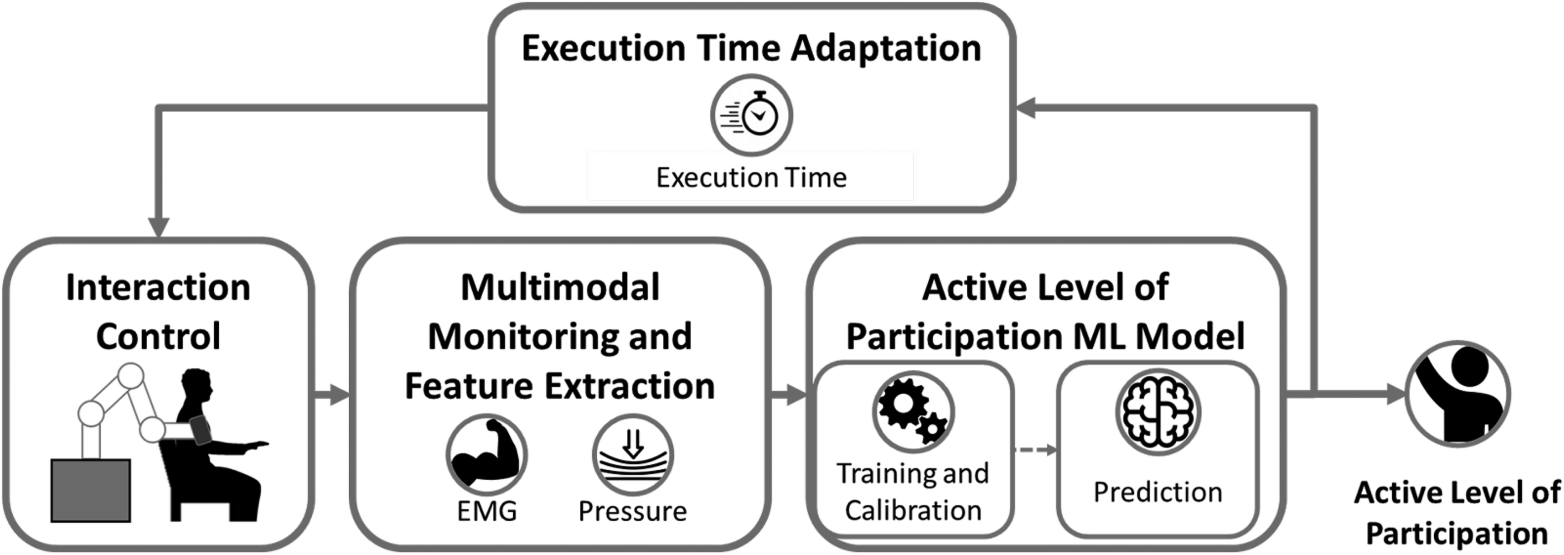
Block diagram of the proposed framework for estimating patient Active Level of Participation (ALP) in robot-aided rehabilitation. The system integrates interaction control, a multimodal monitoring interface (capturing EMG and pressure signals), and a machine learning (ML) model for ALP estimation, which includes training, calibration, and real-time prediction. The estimated ALP is used to dynamically adapt the task execution time, creating a closed-loop control strategy to encourage active patient participation.

Figure 1 presents the block scheme of the proposed approach. During an upper-limb robot-aided rehabilitation session, a multimodal monitoring system can be exploited to measure biomechanical and physiological information of the human-robot interaction. In particular, the EMG reflects the user’s motion intention, providing a direct measure of muscle activation and effort. Complementarily, the pressure distribution sensed at the human-robot interface offers insight into the physical interaction between the user and the robot, capturing the forces exerted during task execution. Using both modalities ensures a more comprehensive assessment of the user’s participation, as EMG focuses on internal physiological signals while pressure sensing reflects external interaction dynamics, enabling a holistic understanding of the patient-robot interaction (21). Features can be extracted from the processes monitored by the human-robot interface to extract a meaningful observation of the interaction.

These quantities can be used to train Machine Learning (ML) supervised algorithms to classify whether the patient is participating or not in accomplishing the motor task. Moreover, reliable continuous estimation of the active level of participation (ALP) can be provided via ML model calibration. Thus, an experiment is needed to build up a structured dataset to model ALP by capturing the behavior of healthy participants during their interaction. The computed ALP can be used to adapt the robot’s behavior. In particular, the proposed approach adapts the task execution time according to the ALP. Indeed, the execution time directly reflects the user’s contribution to accomplishing the task. If the patient starts slacking, the robot stops in order to let the user provide enough effort to make the robot continue the movement.

#### 2.1.1 Multimodal monitoring and feature extraction

To facilitate safe and comfortable interactions, a physical interface is established between the human and the end-effector rehabilitation cobot. This interface incorporates an upper-arm orthosis equipped with built-in pressure sensors and EMG electrodes. Its purpose is to ensure accurate positioning of the robot relative to the user’s body and effective transmission of forces and torques. Recognizing the significant influence of interface dynamics, special attention is given to optimizing the robot’s capacity to offer support and comfort during the rehabilitation process (21, 22).

**Figure 2:**
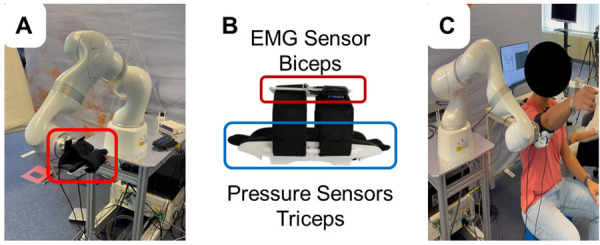
Experimental setup: **(A)** The end-effector robot used for the study. **(B)** Close-up view of the sensorized cuff integrating EMG electrodes (positioned over the biceps) and pressure sensors (positioned near the triceps) to monitor muscle activity and interaction forces. **(C)** A participant performing shoulder flexion/extension movements with robotic assistance.

As depicted in Figure 2, four flexible polymer capacitive pressure sensors are incorporated along the orthosis’s central axis (23). The pressure sensors enable pressure readings at a rate of 10 Hz with a relative accuracy of 10%. This pressure sensor offers a balance between cost-effectiveness, compactness for integration within the robot’s end-effector, and performance aligned with the requirements of our application. In rehabilitation, human-robot interactions usually involve slow, controlled movements. As a result, very high sampling frequencies and high accuracy are not essential. The aim of the proposed multimodal interface is to detect the user’s pressure distribution during the task execution, rather than fine-grained pressure values. The sensors are calibrated in relation to the robot force sensing at the start of each trial.

EMG signals from the biceps brachii are recorded using a Cometa Mini Wave Infinity system (Cometa Srl, Bareggio, Italy) at a sampling rate of 2 kHz. The biceps brachii muscle was selected for monitoring as it plays a prominent role in various upper limb activities, producing distinctive EMG activation patterns (24). Moreover, the EMG sensor electrodes were seamlessly integrated into the straps of the end-effector orthosis. This made it possible to develop an end-effector capable of monitoring multimodal patient parameters who do not need to wear additional sensors.

To extract meaningful information from the monitored data, a fixed temporal window of 1 s is utilized. The data from the multimodal interface, regardless of the varying sample numbers due to different signal acquisition frequencies, is temporally aligned according to their timestamps. Features are then computed over this standardized 1 s time window in the time domain. From the four channels of the measured pressures, several statistical features can be computed. These features include the mean, standard deviation, minimum, maximum, and mean values of the first and second derivatives. These statistics provide insights into the overall level and dynamics of the pressure signals. From the raw EMG signal, both time and frequency domain features are computed. Time-domain features reflect properties of the signal over time and include the root mean square, average amplitude change, variance, integrated EMG, average energy, wavelength, mean absolute deviation, and logarithmic difference of absolute mean values. Mean and median frequencies, which indicate the central tendencies of the signal’s frequency content, are also considered. A total of 10 features are extracted from the EMG signal, capturing its temporal and spectral characteristics. From the pressure signals, 24 features are computed, encompassing statistical measures and derivatives. Overall, a set of 34 features is derived from both the EMG and pressure signals to capture relevant information for the estimation of the ALP. As already demonstrated in previous studies by (21), the integration of multimodal information led to improvements in classification accuracy.

#### 2.1.2 Active level of participation ML model

The ALP estimation model presented in this paper relies on the training and calibration of a supervised ML model. At first, binary classifiers have to be trained to accurately identify the two extreme conditions, i.e., not participating and highly participating, and then their outputs have to be calibrated to estimate in a continuous manner the ALP (25).

Four different ML algorithms were compared in this study: Linear Discriminant Analysis (LDA), Linear Support Vector Machine (SVM), Logistic Regression (LR), and k-Nearest Neighbors (kNN). LDA enhances class separability through linear feature combinations and is particularly well-suited for cases where data distributions for different classes are approximately Gaussian (26, 27). Linear SVM uses hyperplanes to classify data points by maximizing the margin between classes, a technique grounded in Vapnik’s statistical learning theory, which ensures good generalization properties (28, 29). Logistic Regression is a probabilistic model rooted in generalized linear models (30), estimating binary event probabilities based on input variables through the sigmoid function. Lastly, kNN is a non-parametric method inspired by instance-based learning, classifying data points based on the majority vote of their k nearest neighbors in feature space (31, 32).

The choice of these models was motivated by their robustness, interpretability, and effectiveness with relatively small datasets. More complex approaches, like boosted trees or random forests, often demand larger datasets and may overfit when data is limited.

To create the binary participation classification model, participants are instructed to interact with the robot, exhibiting both maximum participation and complete passivity during the training phase, as outlined in (12). This approach allows for the labeling of data collected from the multimodal interface.

To calibrate the outputs of the binary classifiers and estimate the ALP, the isotonic regression calibration approach was employed in this work (33). This calibration technique maps the posterior probability of the supervised learning models to a monotonically increasing function. More in detail, to minimize the discrepancy between the predicted probabilities ($f_{i}$) and the true target probabilities ($p_{i}$), a calibration function m(⋅) is determined. The calibration process accounts for residuals ($\epsilon_{i}$) to improve the alignment between predicted and observed probabilities. Formally, the calibration problem is defined in Equation (1)

(1) $$\hat{m}=\arg\min_{z}\sum_{i}(p_{i}-z(\;f_{i}){)}^{2}$$

where $\left(f_{i},p_{i}\right)$ represents the i-th sample in a calibration dataset. The goal is to optimize the function z(⋅) such that the calibrated predictions align as closely as possible with the observed true probabilities.

Through a comparative analysis of the supervised ML models mentioned above, the most effective approach in estimating the ALP within the context of robot-aided rehabilitation was identified. This comprehensive evaluation allowed us to select the optimal model that provides an accurate and continuous estimation of the ALP, facilitating personalized and adaptive rehabilitation interventions.

#### 2.1.3 Execution time adaptation

Once an estimation of the ALP is available (the ALP model returns estimation every second), it is possible to take it into account to adapt the robot behavior. The approach proposed in this study aims at reducing the duration of the task according to the ALP of the participants. Influencing the duration of task execution, the movement speed resulted to be increased since the robot has to go through the same number of points recorded in the demonstration phase in a shorter time. Given a demonstration trajectory $x_{\mathrm{demo}}$ composed of N samples, at each iteration, the ALP is used to compute the number of samples Δn to skip in the recorded trajectory $x_{\mathrm{demo}}$ in order to assign a reference set-point to the robot as defined in Equation (2)

(2) $$x_{d}(n+1)=x_{\mathrm{demo}}(n+\Delta n).$$

where $x_{d}$ is the current desired pose and Δn is defined in Equation (3) as follows:

(3) $$\Delta n=\left\{ \begin{array}{cc} 0 & IF\;ALP<ALP_{b}\;\;\\ 1 & IF\;ALP_{b}\leq ALP<\left(ALP_{b}+1\right)/2\;\\ 2 & IF\;ALP\geq \left(ALP_{b}+1\right)/2\;\; \end{array}\right.$$

where $ALP_{b}$ is a subject-specific threshold computed in a baseline recording phase. In this way, the lower the ALP, the lower the Δn, and the higher the time needed to complete the replay of the demonstrated trajectory. If the participant slacks, e.g., $ALP\leq ALP_{b}$, the robot will not move until the model estimates a user $ALP\geq ALP_{b}$. The choice of parameter values (0, 1, and 2) in Equation 3 represents distinct action levels based on ALP, with $(ALP_{b}+1)/2$ evenly dividing ALP $\geq ALP_{b}$. These values were not empirically determined but selected for interpretable adaptation control strategy.

#### 2.1.4 Interaction control

The interaction between the robot and the user is managed by a Cartesian impedance controller around a set point. The robot motion dynamics along with the implemented control law are reported in Equation (4)

(4) $$\left\{ \begin{array}{c} \tau_{c}=B(q)y+C(q,\dot{q})\dot{q}+F_{v}\dot{q}+F_{s}\text{sign}(\dot{q})+g(q)\\ y=J^{†}(q)\cdot K\cdot \tilde{x}=J^{†}(q)\cdot K\cdot \left(x_{d}-x_{a}\right)\; \end{array}\right.$$

where B(q) is the robot inertia matrix, $C(q,\dot{q})$ accounts for Centrifugal and Coriolis effects, $F_{v}$ is the viscous friction torque, $F_{s}\text{sign}(\dot{q})$ is the static friction torque, g(q) is the gravity contribution, q, $\dot{q}$ and $\ddot{q}$ are the robot joint position, angular velocity and acceleration, respectively, $\tau_{c}$ is the torque supplied by the actuators and y is the control law. In particular, $J^{†}=J^{T}(J\cdot J^{T}{)}^{-1}$ is the right pseudo-inverse of the robot Jacobian, K is the diagonal stiffness matrix of the task space and $\tilde{x}=x_{d}-x_{a}$ represents the pose error between the desired pose $x_{d}$ and the current pose $x_{a}$.

In order to acquire the demonstration trajectory to be replayed by the robot in a specific session, the robot was set transparent by defining the current stiffness matrix as K=diag{0,0,0,0,0,0} N/m. Once the recording starts, the users can freely move their arm attached to the robot end-effector, and the Cartesian position and orientation are saved in the reference demonstration $x_{\mathrm{demo}}$. The set-point $x_{d}$ introduced in the control law in Equation 4 will be $x_{d}\in x_{\mathrm{demo}}$. As already explained, the desired position is taken sequentially from the demonstrated trajectory according to the participant’s estimated ALP, see Section 2.1.3.

Impedance control and trajectory recording with patient involvement are crucial safety measures in robot-aided rehabilitation. Impedance control adapts the robot’s response to user-generated forces generating compliant physical interactions. Recording trajectories with the patient connected to the robot ensures that robot movements align with the patient’s abilities and natural joint motions, enhancing safety during therapy.

### 2.2 Experimental validation

To validate the proposed approach, an experiment was performed by enrolling 15 healthy participants (10 males and 5 females, 34.5±14.2 mean age). All of them signed a written consent to participate in this study and the study was approved by the Ethical Commission of the UZ Brussel (BUN: 1432022000180). Specifically, the validation of the proposed approach was structured into two experimental sessions. The former session focused on collecting data from human-robot interactions to train and calibrate the machine-learning model for ALP estimation. After the model was validated offline, the latter session implemented the trained model in real-time. During this phase, ALP predictions were timely performed by processing data collected from the custom-developed multimodal interface. The interface extracted features from EMG signals and pressure data, which were provided as input to the trained model to infer the ALP. This enabled the adaptive ALP-driven impedance controller to dynamically modify the task execution time based on the participants’ estimated ALP, providing closed-loop feedback. The second phase aimed to evaluate the differences in performance and engagement achieved with the adaptive ALP-driven controller compared to a conventional impedance controller, highlighting the benefits of tailoring robot behavior to user participation.

Figure 2 presents the experimental setup used in this study. The Kuka iiwa LBR Med, reported in Figure 2A (34), is the robotic device used to deliver motor therapy along with the multimodal monitoring (see Figure 2B). To record the reference trajectory $x_{\mathrm{demo}}$, the subjects are securely strapped into the human-robot interface (see Figure 2C), and their arm’s motion is recorded while the robot is attached. A demonstration is initiated by the subject using a pedal, and the motion capture begins. The participant moves its arm, and the Cartesian poses, including position and orientation, of the end-effector, are recorded. These recorded poses serve as the reference trajectory and are subsequently replayed during the experimental sessions. The end-effector follows the recorded trajectory, allowing the subject to experience the previously demonstrated motion during the rehabilitation exercises. A video providing an accessible overview of the platform used for validating the proposed control strategy can be found online.^1^

In the first experiment, five participants were asked to perform shoulder flexion/extension (sFE) movements with the robot aid. sFE involves lifting the arm forward and lowering it to the side of the body. This motion was chosen due to its relevance in rehabilitation exercises, particularly for training shoulder joint mobility. Moreover, participants were instructed to maintain an extended elbow to prevent multi-joint interactions. Participants comfortably sat near the rehabilitation cobot and inserted their right arm into the human-robot interface for the study. Initially, robots recorded the sFE movement ($x_{\mathrm{demo}}$) while they were transparent to participants’ actions. The stiffness matrix of the robot was set at K=diag{500,500,500,300,300,300} N/m for position and N/rad for orientation, which are typical values for upper limb rehabilitation platforms (35). The participants then completed 30 repetitions of the recorded motion in two experimental conditions: Passive Participation (PP), where they relaxed while the robot guided their arm, and Active Participation (AP), where they actively tried to follow the trajectory and engage in the task.

**Figure 3:**
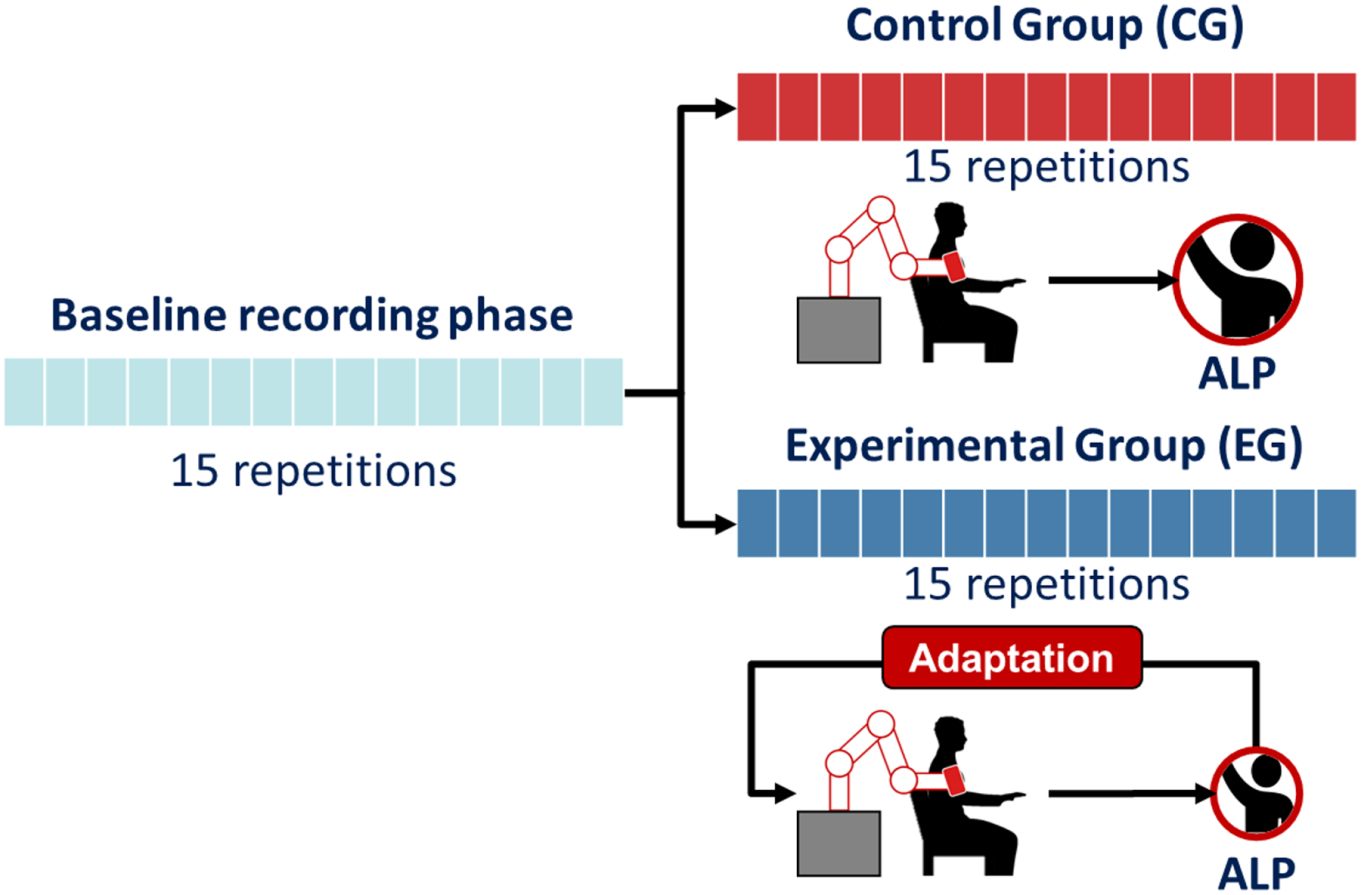
Schematic representation of the protocol for the second experimental validation phase. Both the Control Group (CG) and Experimental Group (EG) performed 15 repetitions during the baseline recording phase. Following this, the CG completed 15 repetitions without robot adaptation, while the EG completed 15 repetitions with the adaptive ALP-driven impedance control.

During the second phase of experimental validation, real-time ALP estimation was utilized to adapt the robot’s behavior. Two groups, each consisting of five participants, were involved in this study. All participants performed 30 repetitions of the sFE task, and no additional information was provided to ensure unbiased results. The experiment began with 15 repetitions to establish a baseline for participant ALP, computed using a subject-specific threshold, $ALP_{b}$, as the average ALP during this phase. For the remaining experiments, a double-blinded approach was adopted, with both the participants and the researcher unaware of which controller was used. The CG interacted with a non-adapting robot with fixed Δn=2 samples, while the EG received rehabilitation using the ALP-adapting robot. A schematic representation of the second experimental session is provided in Figure 3.

### 2.3 Performance indicators

The first experimental phase assesses the ML models capability to estimate the ALP. A k-fold cross-validation (k=5) is carried out on the collected dataset to compute the performance of the implemented classifiers in accurately predicting both the label and the score. In particular, the training set, per each fold, is composed of the 80% of the dataset, and the i-th fold (20%) is split up into a validation set (10%), used to calibrate the classifiers, and a test set (10%), on which are computed the accuracy and the Brier Score (BS). Accuracy is defined as the proportion of the correct predictions with respect to the total number of tested samples. BS measures the accuracy of probabilistic predictions (36) and it can be computed as reported in Equation (5)

(5) $$BS=\frac{1}{N}\sum_{i=1}^{N}{\left(\;f_{i}-o_{i}\right)}^{2}$$

where N is the number of tested samples, $f_{i}$ is the predicted value and $o_{i}$ is the observed one. The BS computes the mean squared error between the predicted probabilities and the observed values. The lower the BS, the better the predictions are calibrated. The best-performing model among the tested ones, e.g., LDA, SVM, LR, and kNN, will be run in the second experimental phase as the ALP model in real-time.

In the second experimental phase, the ALP-adapting robot is compared with respect to the not-adapting condition by means of a set of performance indicators assessing the biomechanics of the human-robot interaction as well as the subjective perception of the controllers.

• ALP: since the underlying hypothesis focuses on investigating whether the real-time adaptation of the behavior of the robot according to the participant’s ALP can indeed foster greater participation, ALP has to be used as a performance indicator.
• Robot mechanical work ($W_{R}$): it serves as an indicator of the user’s contribution to the system. Equation (6) reports the formula to compute the robot mechanical work.
  (6) $$W_{R}=\sum_{t=1}^{T}\left(F(t)\cdot v(t)\cdot \Delta t\right)$$
  where T is the number of samples collected in the experiment, and F and v are the interaction force and the end-effector speed in Cartesian space computed at the time instant t, respectively. The numerical integration of this dot product yields the work done by the robot. Positive work indicates that the interaction force is aligned with the desired motion, while negative work suggests resistance to the robot’s movement. $W_{R}$ is specifically computed during the shoulder flexion phase, where participants actively compensate for the gravitational force.
• Trajectory Error (TE): the error in following the reference position is computed as reported in Equation (7)
  (7) $$TE=\frac{1}{T}\sum_{t=1}^{T}\|p_{d}(t)-p_{a}(t)\|$$
  where T is the number of samples collected in a session phase and $p_{d}$ and $p_{a}$ represent the desired and current position in Cartesian space at the t-th time stamp. The higher the user performance, the lower the TE.
• Integrated EMG (iEMG): since the proposed ALP-adapting robot wants to stimulate the participant to play the main role, the movement intention of the participant was measured by extracting the integral of EMG signal. In particular, the EMG was preprocessed by means of a bandpass 4th order Butterworth filter in the range [15–400] Hz. Moreover, the signal was rectified and a zero-lag 100 ms moving average filter was applied to compute the enveloped EMG (EnEMG). The formula to compute the iEMG is reported in Equation (8)
  (8) $$iEMG=\sum_{t=1}^{T}\left(EnEMG(t)\right)$$
  where EnEMG is the enveloped EMG, T represents the total number of samples collected in an experimental phase, and t indexes the t-th sample. iEMG increase denotes a greater muscular contribution in accomplishing the motor task.

In order to highlight the effect of the robot adaptation on the participants, the aforementioned performance indicators computed during the second 15 repetitions were normalized with respect to the values observed in the baseline recording phase as described in Equation (9)

(9) $$\Delta X=\frac{X-X_{b}}{\left|X_{b}\right|}\cdot 100$$

where X is one among $\left\{ALP,\;W_{R},\;TE,\;iEMG\right\}$ and $X_{b}$ is the mean value of the metrics computed in the baseline phase.

Moreover, the subjective perception of the controllers on the participants is assessed by means of questionnaires. The engagement in interacting with the robot was assessed by means of the Self Assessment Mannequin (SAM) questionnaire. The SAM allows the participants to declare their Valence of the response, perceived Arousal, and perceived Dominance evoked by rehabilitation cobot use. Moreover, the NASA-TLX was administered to assess the perceived workload in interacting with the robot in the two experimental conditions. In particular, the participants were asked to rate from 0-10 their experience in terms of Mental Demand (MD), Physical Demand (PD), Temporal Demand (TD), Performance (PER), Effort (EF), and Frustration (FR).

### 2.4 Statistical analysis

To assess the effect of the user ALP-adapting robot use with respect to the simple impedance controller, a statistical analysis has been carried out on the collected data. In particular, the Wilcoxon rank-sum test is performed on the aforementioned performance indicators for the two groups of participants: CG and EG henceforth. This test assesses whether a significant difference exists between the two investigated conditions. The significance level is set at p-value ≤0.05.

## 3 Results and discussions

In the training phase, a total of 1,500 samples were collected from 5 subjects, with each subject contributing 300 samples, corresponding to a 10 s duration for each of the 30 repetitions of the sFE task.

**Figure 4:**
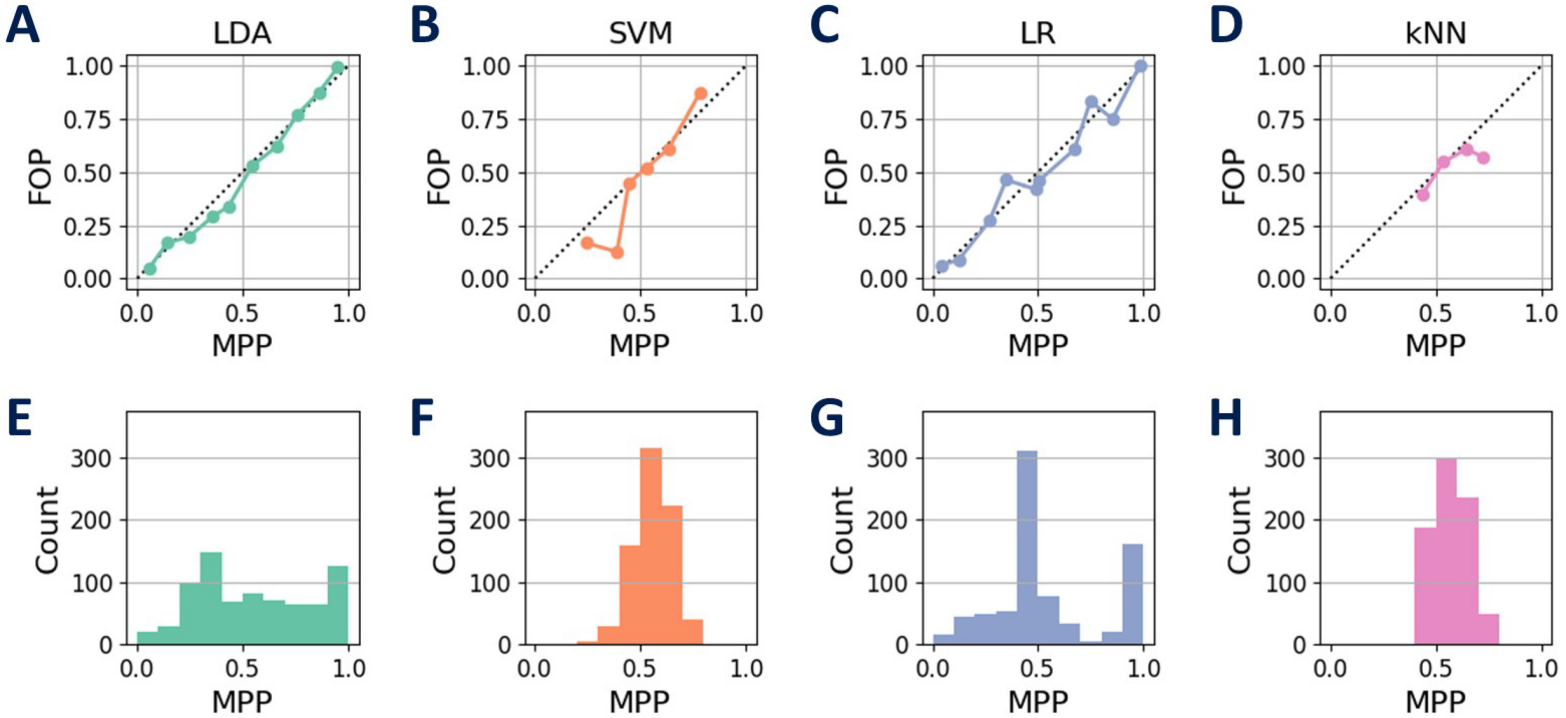
Calibration results of the tested ML approaches. The calibration plots, comparing the mean predicted probabilities (MPP) with the fraction of positives (FOP), are shown for each model: LDA **(A)**, SVM **(B)**, LR **(C)**, and kNN **(D)**. The histograms in panels **(E)**, **(F)**, **(G)**, and **(H)** depict the occurrences of the MPP for each corresponding model. Among the models, LDA demonstrates a calibration closer to a uniform distribution, while the others exhibit a more normally distributed pattern in their calibrated classifiers.

Figure 4 reports the results of the first experimental phase. In particular, the calibration plots, shown in Figures 4A–D, present the mean predicted probabilities (MPP) vs. the fraction of positives (FOP) returned by the four tested ML approaches. The bisector line represents a perfectly calibrated model. Moreover, the histograms of the occurrences of the MPP are reported in Figures 4E–H.

The calibration process aims to refine probability estimations made by ML classifiers. SVM and kNN display less effective calibration, struggling to cover the entire probability range. In contrast, LDA and LR provide more balanced and well-calibrated probabilities. LDA’s unique behavior might arise from its emphasis on class separability, while LR’s statistical nature contributes to uniform distribution. A well-calibrated model should offer balanced predictions for the same MPP. SVM, LR, and kNN result in calibrated classifiers that resemble a normal distribution for MPP, whereas LDA, with less bias toward uniform values, appears closer to a uniform distribution. These differences in performance are influenced by the inherent characteristics of each classifier.

The performance of the tested models is summarized in Table 1. The analysis of the models’ performance shows that LDA achieved the highest accuracy with 75.3±6.9% and the lowest Brier score of 0.15. The other models have a lower performance. These results highlight the distinctive effectiveness of LDA in the specific area of prediction model analysis.

**Table 1:** Calibrated models performance.

	Accuracy [%]	Brier score
Linear discriminant analysis (LDA)	75.3±6.9	0.15±0.01
Support vector machine (SVM)	59.8±4.1	0.23±0.01
Logistic regression (LR)	70.6±5.7	0.17±0.01
k-nearest neighbors (kNN)	58.5±2.6	0.24±0.01

**Figure 5:**
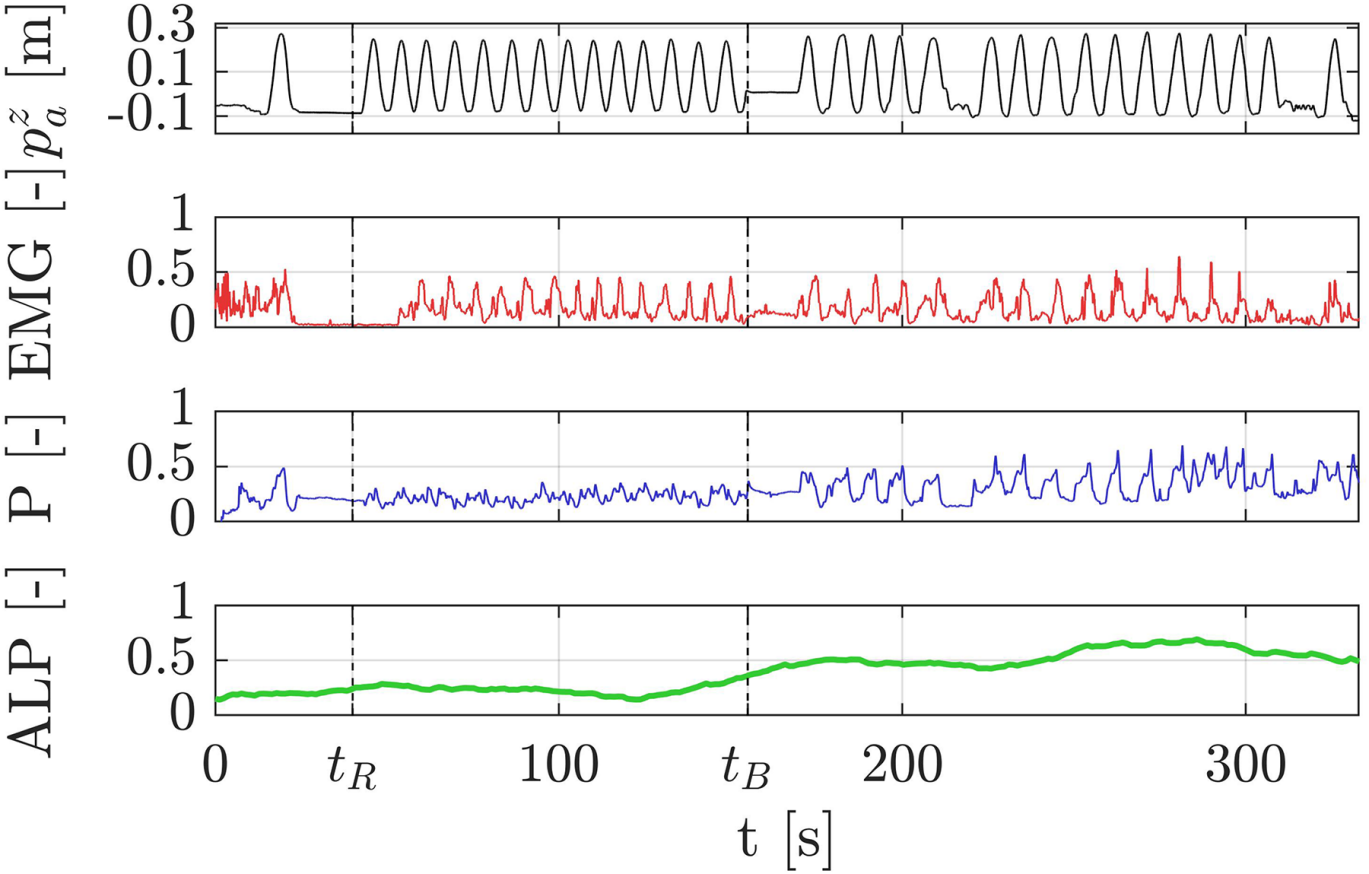
Raw data collected during the second experimental session, including the ALP estimated in real-time for a representative EG participant. The plots display the robot’s position along the *z*-axis ($p_{a}^{z}$), the EMG signals, the normalized pressure data (P), and the estimated ALP. The timeline highlights key phases: demonstration recording ($t\leq t_{R}$), baseline calculation ($t_{R}<t\leq t_{B}$), and the adaptive experimental phase ($t>t_{B}$) where the robot dynamically reacts to ALP. Notably, the participant exhibited an increasing ALP during the adaptive phase compared to the baseline phase. This increase is derived from changes in the EMG and pressure signals, which reflect the participant’s heightened engagement as the robot became responsive to the estimated ALP. This result highlights the effectiveness of the adaptive control strategy in fostering active participation.

In the second experimental session, real-time model accuracy evaluation was hindered because participants were unaware of the experiment’s purpose. They interacted naturally, making binary labels unobtainable. Therefore, the real-time model used data from all training session participants (not subject-specific) to estimate the ALP in the second session. Figure 5 shows the actual position of the robot along the *z*-axis ($p_{a}^{z}$), and the normalized EMG and pressure (P) collected during the second experimental session, along with the ALP estimated in real-time, of a representative EG participant. It’s important to emphasize the temporal sequence: for $t\leq t_{R}$, the demonstration trajectory is recorded, for $t_{R}<t\leq t_{B}$, the baseline is computed, and subsequently ($t\geq t_{B}$), the experimental phase commences where the robot adapts based on the ALP. As soon as the adaptive modality begins, the robot stops and waits for more participant intervention before proceeding with trajectory generation.

**Figure 6:**
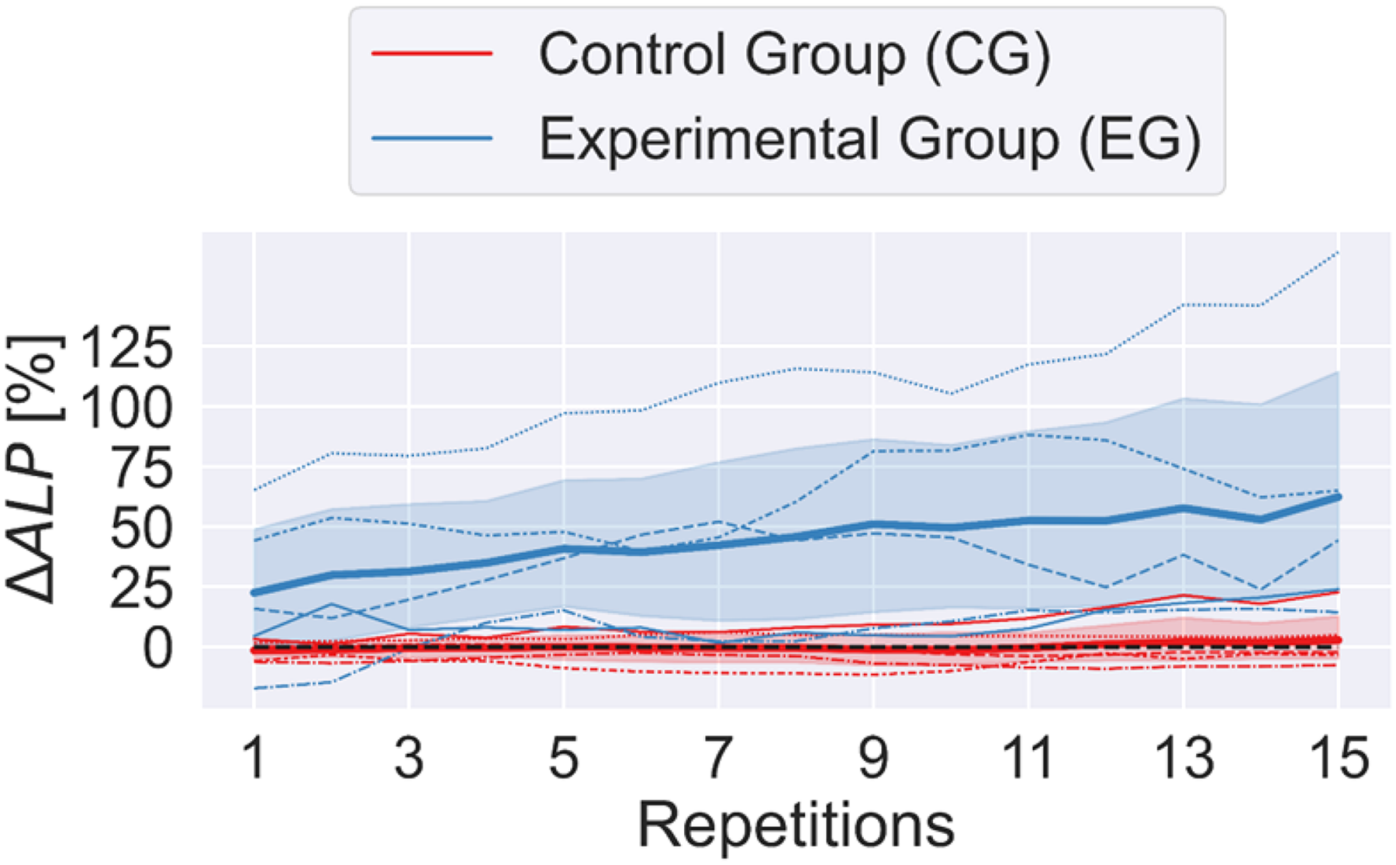
Evolution of ΔALP over 15 experimental repetitions for the Control Group (CG) and Experimental Group (EG). Solid lines represent the mean ΔALP values for each group, with shaded areas indicating 95% confidence intervals. Dashed lines display individual participant ΔALP trends. The CG group exhibited a constant ΔALP, whereas the EG group demonstrated a steady increase, reflecting enhanced active participation.

The ΔALP estimated during the last 15 repetitions of the experiment for the two groups is reported in Figure 6 over time. In particular, the solid lines represent the mean value per each group and the shaded areas stand for the 95% confidence interval. It is worth evidencing that the CG did not exhibit any modification of the ALP over time since the behavior of the robot did not change. Moreover, in order to provide a detailed view of the interaction of the various participants, the dashed lines show the ΔALP estimation of each participant. Examining the ALP estimates of individual participants, notable observations emerge: all the CG participants experienced no significant change in their ALP throughout the experiment. Conversely, EG participants displayed a clear and consistent increasing trend in their ΔALP over time, except for two subjects whose ΔALP remained ≤25% by the end of the session.

**Figure 7:**
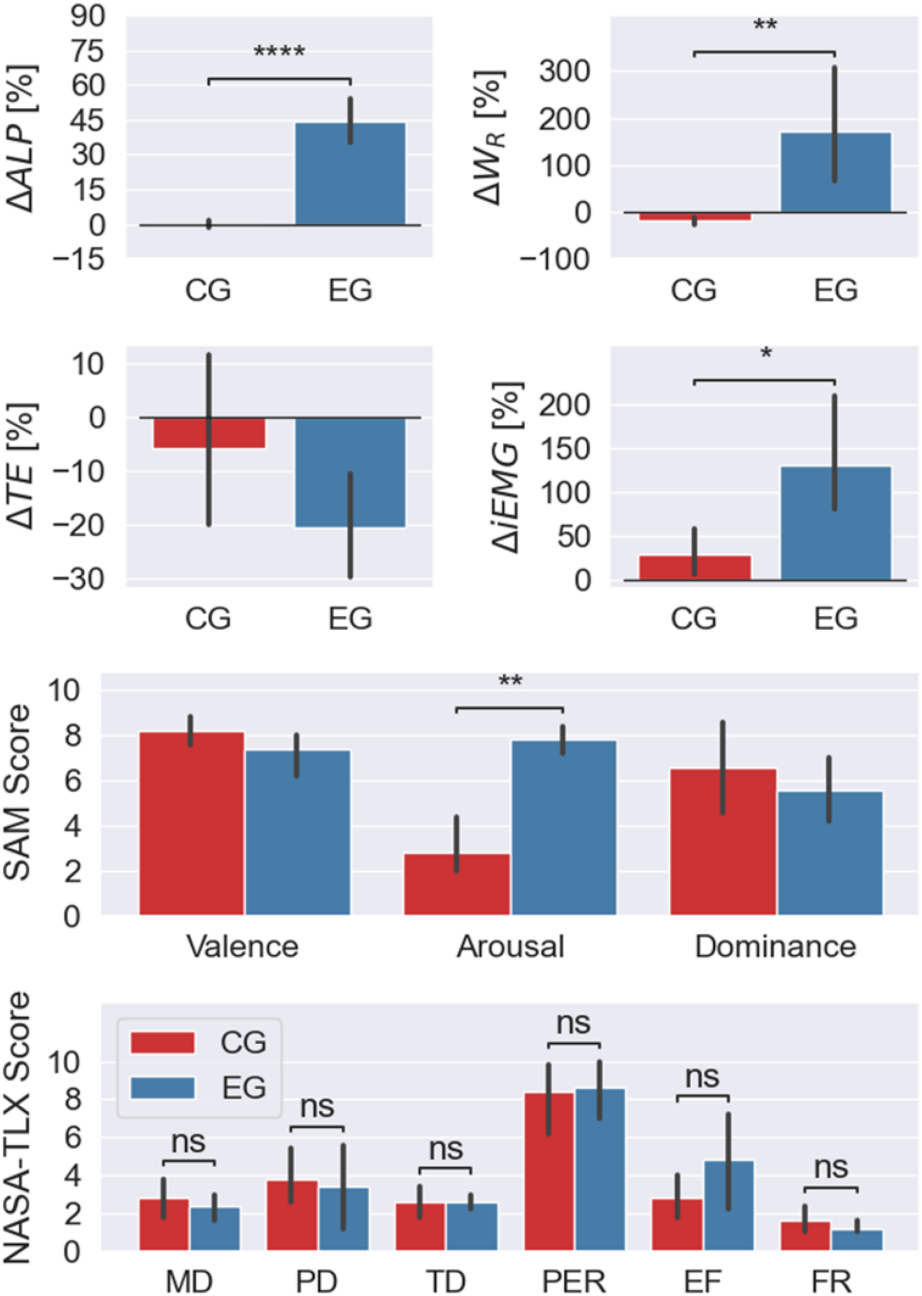
ormance indicators and questionnaire scores (SAM and NASA-TLX) for the two experimental groups. Bars represent the mean values, and solid black lines denote the 95% confidence intervals. Significant differences are observed in ΔALP, $\Delta W_{R}$, ΔiEMG, and SAM Arousal between the Control Group (CG) and Experimental Group (EG), highlighting that the adaptive ALP-driven impedance control effectively increased participation, physical workload, and muscle activation in the EG. The NASA-TLX results showed comparable perceived workload between groups, with a higher, yet not statistically significant, effort reported by the EG.

The ΔALP along with the other performance indicators, introduced in Section 2.3, are reported in Figure 7. The bar plots show the mean value of the indicator computed for the two groups of participants and the solid black line stands for the 95% confidence intervals. Moreover, the statistically significant differences are highlighted by means of asterisks: {∗} for 0.01≤ *p*-value ≤0.05, {∗∗} for $1\times 10^{-3}\leq$ *p*-value ≤0.01 and {∗∗∗∗} for *p*-value $\leq 1\times 10^{-3}$.

As already shown in Figure 6, the ΔALP significantly increased in the second part of the experiment only for the participants who interacted with the ALP-adapting robot (EG).

The participants’ physical workload during the shoulder flexion phase of the sFE exercise was assessed using $W_{R}$ to determine their ability to counteract gravitational forces. The change in $W_{R}$, denoted as $\Delta W_{R}$, showed a notable increase in the experimental phase. For the EG, it changed from −32.9±29.1 J during calibration to 4.3±39.1 J in the experimental phase, indicating increased participant effort. In contrast, the CG displayed a slight decrease in $\Delta W_{R}$, with values of −43.5±32.3 J during calibration and −53.0±42.8 J in the experimental phase. This suggests that CG participants progressively slackened their arm movements as the interaction forces during shoulder flexion opposed the intended motion. Conversely, EG participants exerted more effort, pushing in the same direction as the movement.

The performance of the two experimental groups, in terms of TE, was comparable. The errors in tracking the demonstrated path were $(7.3\pm 6.8)\times 10^{-3}$ m and $(5.4\pm 3.7)\times 10^{-3}$ m for the CG and EG respectively. The participants interacting with the ALP-adapting robot exhibited a non-statistically significant improvement in their performance as soon as the robot started reacting to them. In particular, during the flexion of the participants’ shoulders, they were capable of reaching higher positions on their own.

Muscle activity significantly increased in EG participants during the experimental phase compared to the baseline, as reflected by a remarkable approximately 130% rise in ΔiEMG and a significant difference (*p*-value = 0.01) between the two phases. This substantial increase suggests that the adaptive ALP-driven impedance controller effectively encouraged active participation among EG participants. In contrast, CG participants experienced only a marginal rise in muscle activation compared to their baseline measurements.

These results point out the beneficial effect of the ALP-adapting robot to improve muscle engagement. In the context of rehabilitation, in order to effectively training the targeted body area, an increase in muscle activity is required to stimulate strength recovery. Although it is essential that the patient participates actively by providing mechanical work and muscular effort, it would be important to monitor fatigue and avoid overexertion. The proposed multimodal monitoring interface is a modular platform that offers the potential to integrate additional metrics, such as conduction velocity to better fatigue phenomena (37) and/or add further physiological signals that could enable the detection of inter-individual or gender-related differences.

Lastly, the results regarding the level of engagement and perceived workload are depicted in Figure 7. The Self-Assessment Manikin (SAM) questionnaire revealed that both experimental groups experienced positive feelings and felt confident while interacting with the robot, as indicated by high values of Valence and Dominance, respectively. However, there was a significant difference in the Arousal ranking between the two groups (*p*-value = 0.007). This indicates that interacting with the adaptive ALP-driven impedance controller significantly enhances the participants perceived excitement during the interaction.

The NASA Task Load Index (NASA-TLX) questionnaire showed that the perceived workload was rated in a comparable manner by the two participant groups. However, a not statistically significant difference in the perception of effort can be observed (*p*-value = 0.31). The CG rated the effort perception as 2.8±1.5, while the EG rated it as 4.8±3.0. This may suggest that the ALP-adapting robot was perceived more effort from the participants to proceed with the therapy than the non-adapting robot.

Comparative analysis with a conventional impedance controller underscored the importance of adapting to the individual user’s needs for optimizing robot-aided rehabilitation. The experimental validation yielded promising results, with the adaptive ALP-driven impedance control effectively modifying task duration based on user ALP. While both CG and EG achieved accurate trajectory tracking, EG participants displayed performance improvement and increased muscle activation. Subjective feedback reflected positive experiences and confidence in robot interaction, with the adaptive controller significantly heightening perceived excitement.

While our study involved 10 participants (divided into control and experimental groups), it aligns with the sample sizes reported in most studies exploring innovative adaptive control strategies in robotic rehabilitation, which primarily use healthy participants. For instance, (13) tested their multi-sensor system with 12 healthy volunteers, (14) validated their cooperative control strategy with 4 healthy participants, (38) conducted preliminary tests with 3 healthy individuals, and (39) included 12 healthy participants and preliminary tested their proposed approach with 2 post-stroke patients. This trend highlights that the majority of studies focus on healthy participants in their initial evaluations, as we did, to demonstrate the feasibility and technological effectiveness of their methods.

Moreover, it is worth noticing that the proposed method has the potential to be further enriched by incorporating data from individuals exhibiting non-physiological motor behaviors, such as those resulting from impairments or pathologies. Integrating such behaviors into the training dataset would enhance the model’s capacity to generalize, allowing it to accurately estimate ALP for a wider range of users, including those with motor dysfunctions.

Lastly, a limitation of this study that must be acknowledged is that the ALP-based control strategy was solely validated in a controlled laboratory setting against a conventional impedance control approach. While the results underscore the potential of the proposed methodology to enhance user active participation, it is worth extending this validation to clinically relevant settings. It is recommended that subsequent studies concentrate on evaluating the clinical efficacy of the ALP-guided control strategy by involving patients with motor disabilities and assessing its impact on functional recovery and therapeutic outcomes in real-world rehabilitation settings.

## 4 Conclusion

This paper introduced a novel methodology for objectively estimating the ALP in upper-limb robot-aided rehabilitation sessions and an adaptive ALP-driven impedance controller. The estimation model exploits information coming from a multimodal monitoring interface that captures electromyographic activity and pressure at the human-robot interface.

A machine-learning model training and calibration procedures were performed using data collected from five healthy participants, with LDA identified as the most suitable classifier based on its accuracy and calibration quality. The calibrated model was then used to estimate real-time ALP in two groups of five participants each: CG and EG, interacting with a rehabilitation cobot. The EG participants, experiencing the proposed adaptive ALP-driven impedance controller, exhibited significantly higher physical workload, muscle activity, and perceived excitement compared to the CG. These findings indicate that closing the robot control loop effectively enhances interaction and stimulates participants to increase their level of participation.

Future work will focus on clinically validating this approach with a larger and more diverse participant population, including individuals with limited motor functions, to strengthen the statistical significance of the promising results observed in this study. Additionally, efforts will be devoted to integrating additional sensors for enhanced monitoring of patient parameters. Moreover, estimating ALP could assist in quantifying patients’ participatory abilities at various stages of their motor recovery by integrating ALP with conventional medical assessment scales.

## Data Availability

The original contributions presented in the study are included in the article/Supplementary Material, further inquiries can be directed to the corresponding author.

## Footnotes

^1^The proposed system was validated using the SAFER robotic platform. The SAFER platform was first recognized as a finalist in the KUKA Innovation Award 2022 “Robotics in Healthcare Challenge,” highlighting its potential in rehabilitation robotics. Following this achievement, the system was showcased for a week at the MEDICA Trade Fair 2022. https://www.youtube.com/watch?v=7mKhciQYCus.
